# Autophagy regulated by the HIF/REDD1/mTORC1 signaling is progressively increased during erythroid differentiation under hypoxia

**DOI:** 10.3389/fcell.2022.896893

**Published:** 2022-08-24

**Authors:** Jian Li, Cheng Quan, Yun-Ling He, Yan Cao, Ying Chen, Yu-Fei Wang, Li-Ying Wu

**Affiliations:** State Key Laboratory of Proteomics, Beijing Proteome Research Center, Beijing Institute of Radiation Medicine, Beijing, China

**Keywords:** erythroid differentiation, hypoxia, autophagy, HSPCs, K562, HIF-1, REDD1

## Abstract

For hematopoietic stem and progenitor cells (HSPCs), hypoxia is a specific microenvironment known as the hypoxic niche. How hypoxia regulates erythroid differentiation of HSPCs remains unclear. In this study, we show that hypoxia evidently accelerates erythroid differentiation, and autophagy plays a pivotal role in this process. We further determine that mTORC1 signaling is suppressed by hypoxia to relieve its inhibition of autophagy, and with the process of erythroid differentiation, mTORC1 activity gradually decreases and autophagy activity increases accordingly. Moreover, we provide evidence that the HIF-1 target gene REDD1 is upregulated to suppress mTORC1 signaling and enhance autophagy, thereby promoting erythroid differentiation under hypoxia. Together, our study identifies that the enhanced autophagy by hypoxia favors erythroid maturation and elucidates a new regulatory pattern whereby autophagy is progressively increased during erythroid differentiation, which is driven by the HIF-1/REDD1/mTORC1 signaling in a hypoxic niche.

## Highlights

Hypoxia dramatically accelerates erythroid differentiation.
Autophagy is enhanced by hypoxia during erythroid differentiation.
mTORC1 activity gradually decreases and autophagy activity increases accordingly during erythroid differentiation.
Autophagy is progressively increased with erythroid differentiation, which is controlled by the HIF-1/REDD1/mTORC1 signaling under hypoxia.

## Introduction

A growing body of evidence suggests that adult hematopoietic stem and progenitor cells (HSPCs) reside in the hypoxic bone marrow microenvironment (also termed as niche), commonly referred to as a “hypoxic niche” (Eliasson and Jonsson, 2010; Mohyeldin et al., 2010; Mendelson and Frenette, 2014; Morrison and Scadden, 2014), which controls the fate of HSPCs. Many studies have demonstrated the roles of hypoxia in preserving quiescence, self-renewal, and in promoting proliferation of HSPCs (Cipolleschi et al., 1993; Mohyeldin et al., 2010; Mendelson and Frenette, 2014), but the effect of hypoxia on HSPC differentiation is poorly understood. Although a few studies have shown that hypoxia can regulate erythropoiesis (Haase, 2010; Watts et al., 2020), the effect of ‘hypoxic niche’ on erythroid differentiation and the underlying mechanisms are currently unclear.

During erythroid differentiation, macroautophagy (hereafter referred to as autophagy) plays a critical role in eliminating organelles (Kundu et al., 2008; Griffiths et al., 2012; Zhang et al., 2015; Cao et al., 2016; Grosso et al., 2017). In particular, clearance of mitochondria, termed as mitophagy, is absolutely important for the maturation of red blood cells (RBCs) at the terminal stage of erythroid development (Schweers et al., 2007; Chen et al., 2008; Kundu et al., 2008; Sandoval et al., 2008; Mortensen et al., 2010; Davuluri et al., 2016; Grosso et al., 2017; Kuhikar et al., 2020; Moras et al., 2020; Moras et al., 2021). Autophagy is a highly conserved metabolic process, which can be promoted under various stresses to improve cell survival or to meet developmental demands (Kuma et al., 2004; Levine and Klionsky, 2004; Lum et al., 2005; Zhang and Ney, 2008; Dikic and Elazar, 2018; Allen and Baehrecke, 2020). In addition to removing damaged or unwanted organelles or proteins, autophagy can quickly provide recyclable nutrients such as amino acids, nucleic acid, free fatty acids and ATP for stress or development by degrading metabolic macromolecular substrates (Levine and Klionsky, 2004; He and Klionsky, 2009; Filomeni et al., 2015; Wang et al., 2016). Autophagy can be specifically induced by stress or developmental signals. Although autophagy plays a vital role in mitochondrial clearance at the stage of terminal erythroid differentiation, the role of autophagy at other stages of erythroid differentiation, especially in a hypoxic niche, is almost completely unknown. Therefore, it is necessary to clarify whether autophagy is required at other stages of erythroid development besides the terminal stage, particularly under hypoxia.

The key upstream regulators of autophagy include the mammalian target of rapamycin complex 1 (mTORC1) and the adenosine monophosphate-activated protein kinase (AMPK) signaling pathways. mTORC1, a central regulator in the induction of protein synthesis, is a key switch that controls autophagy through the inhibitory phosphorylation of Unc-51 like autophagy activating kinase 1 (ULK1) complex which is required for initiating autophagy. The activation of mTORC1 switches off autophagy to meet the normal needs of cell growth, while the inactivation of mTORC1 switches on autophagy in response to cell stress or to meet the special demands for development (Ka et al., 2017; Schmeisser and Parker, 2019; Xiang et al., 2019; Zhu et al., 2020). Recently, a study has shown that mTOR inhibition with rapamycin (Rapa) dramatically promotes enucleation and mitochondrial clearance by enhancing autophagy during the maturation phase of erythropoiesis (Liu et al., 2020). However, it has not yet elucidated how mTOR signaling is regulated in any physiological or pathological conditions. Under hypoxia, mTORC1 is inactivated by hypoxia inducible factor 1 (HIF-1), a master regulator of cell response to hypoxia (Bohensky et al., 2010; Kucejova et al., 2011; Zhao et al., 2012; Blagosklonny, 2013; Kuwagata et al., 2016), via the two different pathways. In one pathway, the HIF-1 target gene BCL2 interacting protein 3 (BNIP3) interacts with Rheb, a small GTPase that can activate mTORC1, resulting in a decrease in mTORC1 activity (Li et al., 2007; Mesquita et al., 2020). In the other pathway, the HIF-1 target gene DNA damage inducible transcript 4 (DDIT4/REDD1) competitively binds with 14-3-3 to release TSC complex subunit 2 (TSC2), a GTPase-activating protein (GAP), which negatively regulates the activity of Rheb, from the 14-3-3/TSC2 complex, thereby inactivating mTORC1 (Brugarolas et al., 2004; Hoppe-Seyler et al., 2017; Sun and Yue, 2019; Gao et al., 2020; Shang et al., 2020). AMPK functions as a central mediator of the cellular response to energetic stress. Prolonged hypoxia eventually leads to energy deficiency and AMPK activation. Once activated, AMPK can induce autophagy via two pathways: ULK1 is directly activated by AMPK to form a complex with ATG13, ATG101 and FIP200 to initiate autophagy (Liu et al., 2010; Han et al., 2021; Mei et al., 2021; Trelford and Di Guglielmo, 2021); mTORC1 is inactivated by AMPK through independently phosphorylating the mTORC1 upstream negative regulator TSC2 or the mTORC1 subunit RAPTOR, relieving the inhibitory phosphorylation of mTORC1 and hence switching on autophagy (Seto-Tetsuo et al., 2021; Xie et al., 2021). So far, whether autophagy regulates the erythroid differentiation under hypoxia and which pathways are involved in the induction of autophagy in this context have not been elucidated yet.

In the current study, we used the human erythroleukemia cell line K562 and human primary umbilical cord blood (UCB)-derived HSPCs to demonstrate that hypoxia can accelerate erythroid differentiation and enhance autophagy in this process. We determined the role of autophagy in accelerating erythroid differentiation under hypoxia. Through transcriptomic analysis and identification of genes related to hypoxia response during erythroid differentiation, we uncovered that the HIF-1/REDD1/mTORC1 axis plays a crucial role in inducing autophagy, which promotes the erythroid differentiation under hypoxia. Moreover, we elucidated the role of mTORC1 in controlling erythroid differentiation by regulation of autophagy, in addition to by regulation of mitochondrial biosynthesis, and identified a new regulatory pattern of erythroid differentiation under the control of mTORC1 and autophagy in normoxia and hypoxia conditions. Thus, our study provides an understanding of the role of hypoxic niche in erythropoiesis and its physiological and pathological significance, which may have important implications for the treatment of erythropoietic disorders.

## Results

### Hypoxia promotes erythroid differentiation

**FIGURE 1:**
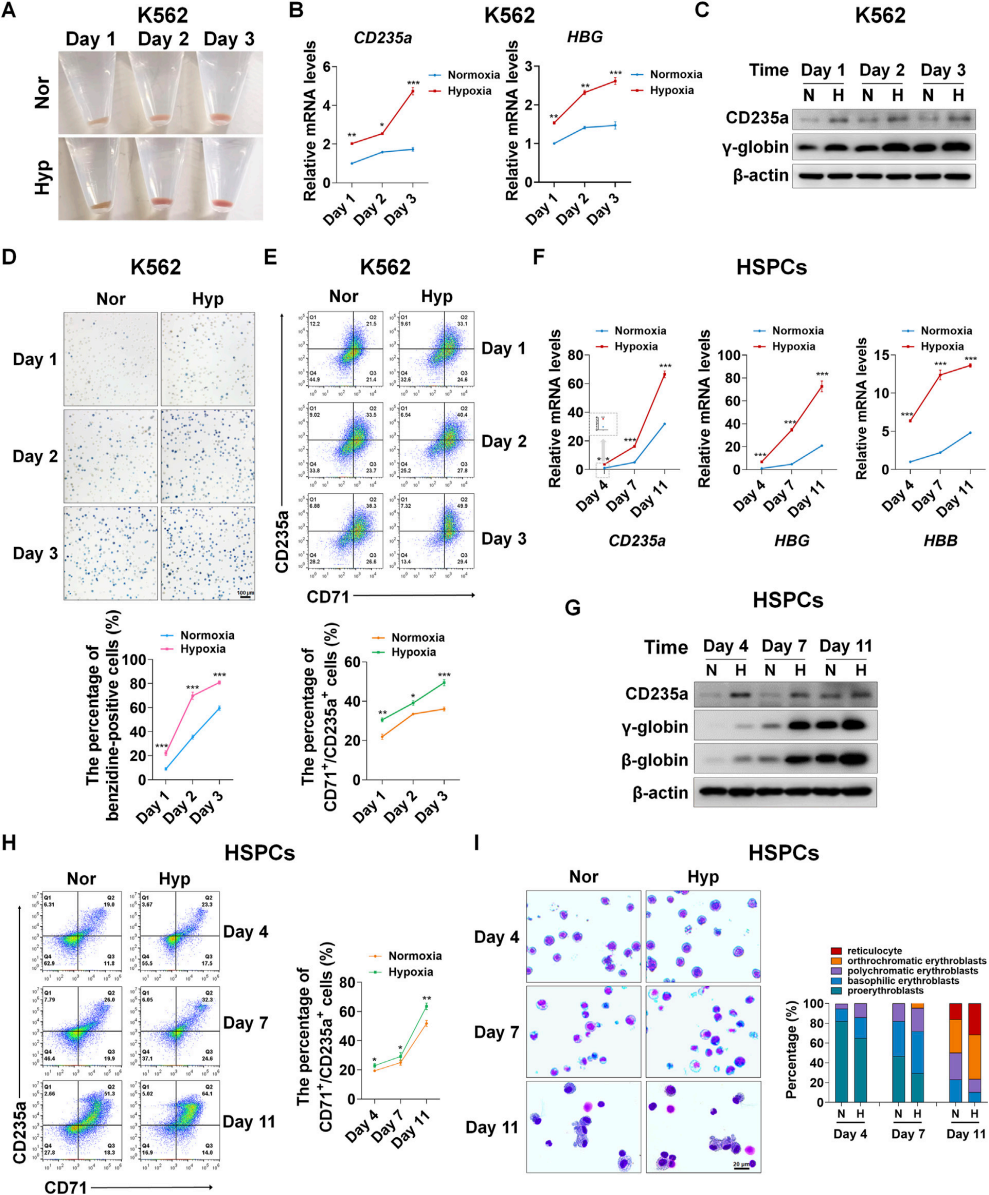
Hypoxia accelerates erythroid differentiation **(A)** Reddish pellets of K562 cells in the process of erythroid differentiation under normoxia and hypoxia. Redder pellets indicate more accumulation of hemoglobin **(B)** qRT-PCR analysis of the relative mRNA levels of specific erythrocyte markers CD235a and HBG. qRT-PCR was performed at the indicated time points after K562 cells were exposed to normoxia or hypoxia during erythroid differentiation. *p < 0.05, **p < 0.01, ***p < 0.001, versus the corresponding normoxia group at different time points **(C)** Western blot analysis of CD235a and γ-globin protein expression at the indicated time points after K562 cells were exposed to normoxia or hypoxia. N: normoxia; **(H)** hypoxia **(D)** Representative images of benzidine staining in K562 cells differentiated into erythrocytes. The line graph below shows the percentage of benzidine-positive cells. Scale Bar = 100 μm ***p < 0.001, versus the corresponding normoxia group. Nor: normoxia; Hyp: hypoxia **(E)** Flow cytometry plots show the expression of CD235a and CD71 in K562 cells. The line graph below shows the percentage of CD71^+^/CD235a^+^ cells. *p < 0.05, **p < 0.01, ***p < 0.001, versus the respective normoxia group **(F)** qRT-PCR analysis of the relative mRNA levels of CD235a, HBG and HBB in HSPCs. qRT-PCR was performed at the indicated time points after HSPCs were exposed to normoxia or hypoxia during erythroid differentiation. ***p < 0.001, versus the respective normoxia group **(G)** Western blot analysis of CD235a, γ-globin and β-globin protein expression at the indicated time points after HSPCs were exposed to normoxia or hypoxia during erythroid differentiation. N: normoxia; H: hypoxia **(H)** Flow cytometry plots show the expression of CD235a and CD71. The line graph on the right shows the percentage of CD71^+^/CD235a^+^ cells. ns. not significant, *p < 0.05, **p < 0.01, versus the respective normoxia group **(I)** May-Grunwald Giemsa staining of HSPCs exposure to normoxia or hypoxia for different periods of time during the process of differentiation into erythrocytes. Representative images were shown for proerythroblasts, basophilic erythroblasts, polychromatic erythroblasts, orthrochromatic erythroblasts and reticulocytes. Scale Bar = 20 μm. The right panel shows the morphologic classification of erythroblasts. Cell types were determined by examining 200 cells per group and expressed as a percentage.

Little is known about the regulation of hypoxia on erythroid differentiation. In view of this, first of all we determine the effect of hypoxia on erythroid differentiation using K562 cells and human UCB-derived HSPCs, respectively. K562 cells were induced to differentiate into erythroid cells with hemin for 1, 2, and 3 days. The color of the pellets was first observed to preliminarily assess the ability to differentiate into erythroid cells. With the time of differentiation, the pellets gradually turned red, and compared with normoxia, hypoxia darkened the red pellets (Figure 1A), indicating that more erythroid cells were generated under hypoxia conditions. Real-time PCR and western blot analysis of the expression of CD235a and γ-globin representing the erythroid differentiation showed that hypoxia significantly increased their mRNA (*CD235a* and *HBG*) and protein expression levels (Figures 1B,C), suggesting that hypoxia can promote erythroid differentiation. K562 cells from day 1, 2, 3 of differentiation were subsequently stained with benzidine to detect hemoglobin biosynthesis that means differentiation into erythroid cells. The benzidine staining illustrated that the percentage of benzidine-positive cells increased with time, and there were more benzidine-positive cells in hypoxia than normoxia conditions at the corresponding time points (Figure 1D). Flow cytometry analysis of the expression of CD71 and CD235a, which are specific erythroid surface antigens and mainly expressed on relatively early and late erythroid cells, provided further evidence that hypoxia is more conducive to the erythroid differentiation than normoxia (Figure 1E).

In accordance with the phenotypic changes in K562 cells, in HSPCs derived from human umbilical cord blood, we observed a more powerful role of hypoxia in promoting erythroid differentiation. HSPCs were induced to differentiate into erythroid cells using the established culture system with minor modifications (Zheng et al., 2014), and the erythroid differentiation was monitored on day 4, 7, and 11. The erythroid differentiation was assessed by the expression of specific erythrocyte marker molecules. The mRNA levels of *CD235a*, *HBG* and *HBB* increased with the time of differentiation, and notably, their mRNA levels were distinctly higher in hypoxia than normoxia conditions (Figure 1F). Correspondingly, the protein levels of CD235a, γ-globin and β-globin also increased with the time of differentiation, and hypoxia resulted in higher expression levels than normoxia at each time point, especially on the 7th and 11th day of differentiation (Figure 1G). Similarly, the co-expression of CD235a and CD71 analyzed by flow cytometry showed that the percentage of differentiated erythroid cells was increased by hypoxia as expected (Figure 1H). In addition, using May-Grunwald Giemsa staining we found that during erythroid development, hypoxia strongly promoted the maturation of erythroid cells (Figure 1I). Together, the above evidence we provide demonstrates that hypoxia is beneficial to accelerate the process of erythroid differentiation.

### Hypoxia enhances autophagy in the process of erythroid differentiation

**FIGURE 2:**
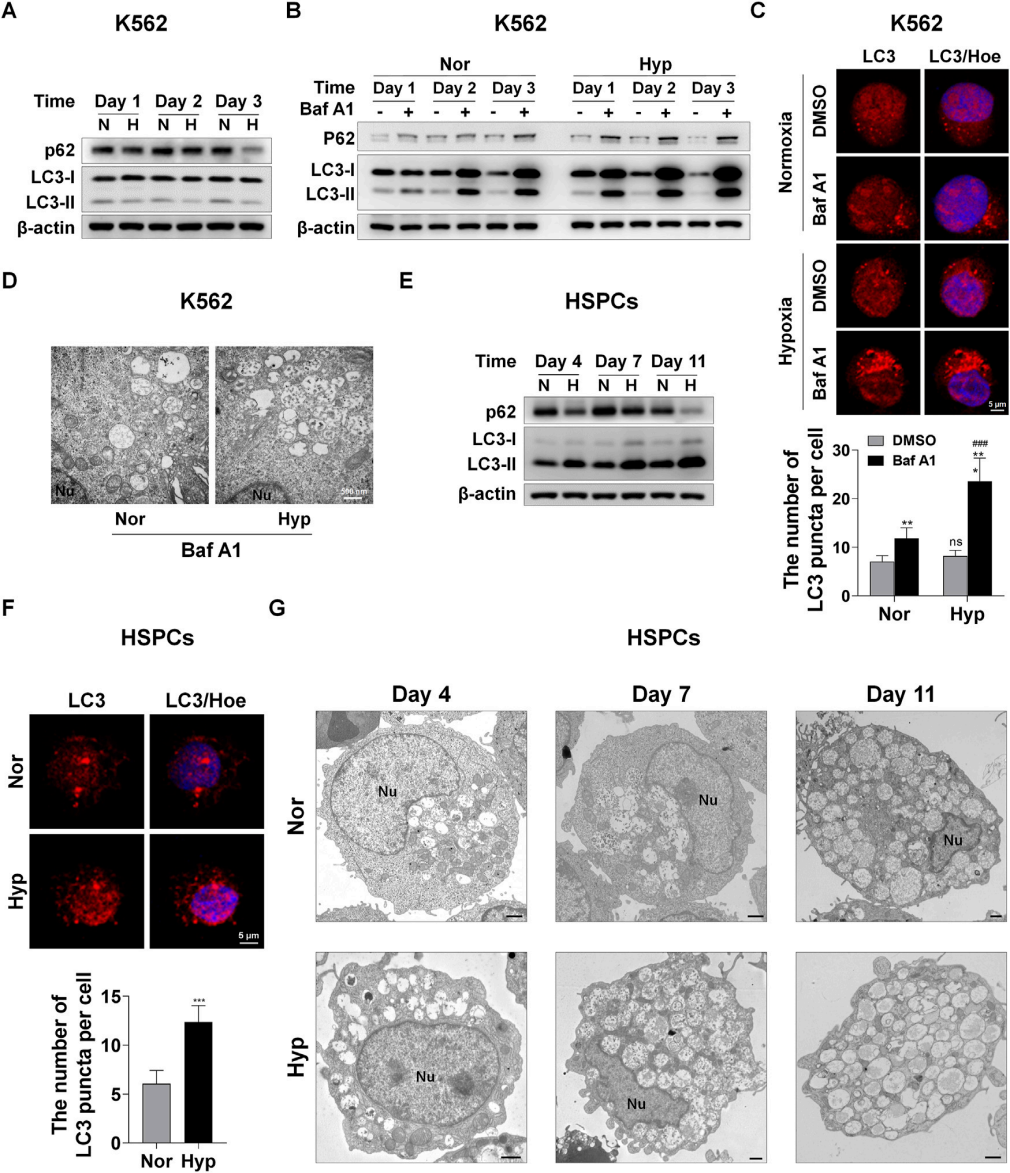
Hypoxia enhances autophagy during erythroid differentiation **(A)** Western blot analysis of p62 and LC3 protein expression at the indicated time points after K562 cells were exposed to normoxia or hypoxia during erythroid differentiation. N: normoxia; H: hypoxia **(B)** Western blot analysis of p62 and LC3 protein expression at the indicated time points after K562 cells treated with DMSO (-) as control or Baf A1 (+, 20 nM) were exposed to normoxia or hypoxia. Nor: normoxia; Hyp: hypoxia **(C)** Immunofluorescence analysis of LC3 puncta in K562 cells differentiated into erythrocytes. K562 cells were treated with DMSO or Baf A1 (20 nM) under normoxia or hypoxia for 48 h. Cell nucleus labeled with hoechst33342 (Hoe) is shown in blue, LC3 staining is shown in red and the merged images display their co-localization. Scale Bar = 5 μm. The graph on the right shows the quantification of LC3 puncta per cell. ns, not significant., ^###^ *p* < 0.001, versus the respective normoxia group. ^**^ *p* < 0.01, ^***^ *p* < 0.001, versus the corresponding DMSO group **(D)** TEM imaging of K562 cells treated with Baf A1 (20 nM) exposure to normoxia or hypoxia for 72 h. Representative images of autophagosomes are shown at 30000X. Nu: nucleus. Scale Bar = 500 nm **(E)** Western blot analysis of p62 and LC3 protein expression at the indicated time points after HSPCs differentiated into erythrocytes were exposed to normoxia or hypoxia. N: normoxia; H: hypoxia **(F)** Immunofluorescence analysis of LC3 puncta in HSPCs-derived erythrocytes. HSPCs were induced into erythrocytes under normoxia or hypoxia conditions for 7 days. Cell nucleus labeled with hoechst33342 (Hoe) is shown in blue, LC3 staining is shown in red and the merged images display their co-localization. Scale Bar = 5 μm. The graph below shows the quantification of LC3 puncta per cell. ^***^ *p* < 0.001, versus the normoxia group **(G)** TEM imaging of HSPCs-differentiated erythrocytes cultured under normoxia or hypoxia. Nu: nucleus. Scale Bar = 1 μm.

Autophagy is a conserved cellular catabolism mechanism by which the degraded metabolites rapidly provide the basic building blocks needed by a cell for anabolic processes, such as cell growth, proliferation and differentiation (Levine and Klionsky, 2004; Dikic and Elazar, 2018; Allen and Baehrecke, 2020). Given that autophagy can quickly supply blocks/, we wonder whether autophagy is required for the erythroid differentiation accelerated by hypoxia. First, in K562-differentiated erythroid cells, we tested the expression of autophagy receptor protein sequestosome 1 (SQSTM1/p62) and autophagy marker microtubule-associated protein 1 light chain 3 (MAP1LC3B/LC3) via western blot analysis, and found a seemingly paradoxical phenomenon, that is, hypoxia simultaneously reduced the expression of p62 and LC3-II (Figure 2A). Generally, when the protein level of p62 decreases due to autophagy, the LC3-II protein level or the flux of LC3-II/LC3-I increases. To identify whether autophagy is enhanced by hypoxia, we used the late stage autophagy inhibitor bafilomycin A1 (Baf A1) to treat K562-differentiated erythroid cells under normoxia and hypoxia, and then observed that p62 and LC3-II proteins were markedly accumulated by Baf A1 and the accumulation became more with the time of differentiation, especially under hypoxia conditions (Figure 2B), indicating that autophagy increased with the differentiation process, and hypoxia actually enhanced autophagic flux. For ease of detection, the decrease in p62 protein level is used to indicate autophagy in K562 cells in the following experiments. During autophagy, LC3 is processed to form LC3-II, which is then recruited to the outer and inner membranes of the autophagosome. Therefore, cells undergoing autophagy can be identified by visualizing fluorescently labeled LC3 puncta. Likewise, by means of immunofluorescence staining of endogenous LC3 expression and counting the number of puncta, we noticed that the LC3 puncta were not obviously increased by hypoxia exposure, but after Baf A1 treatment, there were more LC3 puncta in hypoxia than normoxia conditions (Figure 2C). Furthermore, we used transmission electron microscopy (TEM) to compare the differences in autophagosomes between normoxia and hypoxia after blocking autophagy with Baf A1 in K562-differentiated erythroid cells. As illustrated in Figure 2D, we observed that more autophagosomes appeared under hypoxia than normoxia, indicating that hypoxia accelerates autophagic flux.

During the erythroid differentiation of HSPCs, we found that hypoxia resulted in an increase in LC3-II and a decrease in p62 through examining protein levels via western blot analysis (Figure 2E). This clearly demonstrates that autophagy increases with the erythroid differentiation and hypoxia augments autophagy in this process. Immunofluorescence analysis of LC3 expression showed that hypoxia stimulated more LC3 puncta formation than normoxia (Figure 2F). At the same time, we used TEM to compare the dynamic changes of autophagosomes in HSPCs-differentiated erythrocytes under hypoxia and normoxia. We were pleasantly surprised to find that the number of autophagosomes was obviously more in hypoxia than normoxia, and increased with differentiation time in both normoxic and hypoxic conditions (Figure 2G). The above results suggest that hypoxia can enhance autophagy which increases with erythroid differentiation.

### Autophagy mediates erythroid differentiation promoted by hypoxia

**FIGURE 3:**
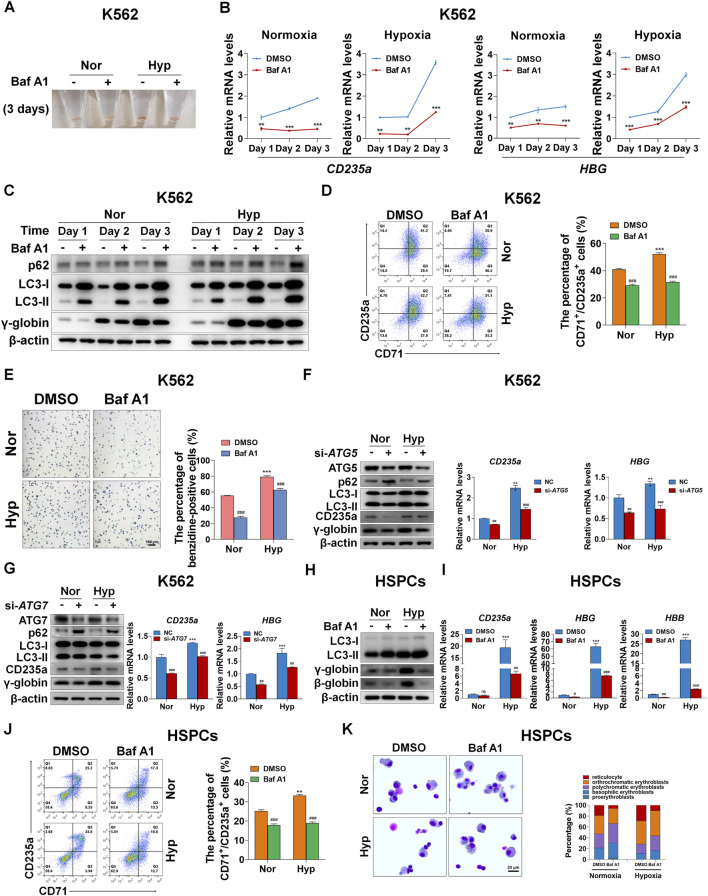
Autophagy mediates erythroid differentiation promoted by hypoxia **(A)** Reddish pellets of K562 cells differentiated into erythrocytes treated with DMSO or Baf A1 (20 nM) under normoxia and hypoxia. The red color of pellets was lightened by Baf A1 in both normoxia and hypoxia **(B)** qRT-PCR analysis of the relative mRNA levels of CD235a and HBG at the indicated time points after K562 cells treated with DMSO (-) as control or Baf A1 (+, 20 nM) were induced into erythroid differentiation under normoxia or hypoxia conditions. **p < 0.01; ***p < 0.001. versus the respective normoxia group at different time points **(C)** Western blot analysis of p62, LC3 and γ-globin protein expression at the indicated time points after K562 cells differentiated into erythrocytes were treated with DMSO (-) as control or Baf A1 (+, 20 nM) under normoxia or hypoxia. Nor: normoxia; Hyp: hypoxia **(D)** Flow cytometry plots show the expression of CD235a and CD71 in K562 cells differentiated into erythrocytes that were treated with DMSO (-) as control or Baf A1 (+, 20 nM) under normoxia or hypoxia for 3 days. The graph on the right shows the percentage of CD71^+^/CD235a^+^ cells. ***p < 0.001, versus the DMSO group under normoxia. ^###^ *p* < 0.001, versus the respective DMSO group under normoxia or hypoxia **(E)** Representative images of benzidine staining in K562 cells treated the same as **(D)**. The right graph shows the percentage of benzidine-positive cells. Scale Bar = 100 μm ***p < 0.001, versus the DMSO group under normoxia. ^###^ *p* < 0.001. versus the respective DMSO group under normoxia or hypoxia. Nor: normoxia; Hyp: hypoxia **(F,G)** The left panel shows the western blot analysis of the effects of ATG5/ATG7 knockdown on p62, LC3, CD235a and γ-globin protein expression in K562 cells differentiated into erythrocytes that were transfected with scramble siRNA as negative control (NC) or si-ATG5/ATG7 under normoxia or hypoxia for 3 days. The two panels on the right show the qRT-PCR analysis of the relative mRNA levels of CD235a and HBG. ***p < 0.001, versus the NC group under normoxia. ^##^ *p* < 0.01, ^###^ *p* < 0.001. versus the respective NC group under normoxia or hypoxia **(H)** Western blot analysis of the effects of Baf A1 (20 nM) on LC3, γ-globin and β-globin protein expression in HSPCs-differentiated erythrocytes which were treated with DMSO or Baf A1 under normoxia or hypoxia for 7 days **(I)** qRT-PCR analysis of the effects of Baf A1 on CD235a, γ-globin and β-globin mRNA expression in HSPCs-differentiated erythrocytes treated the same as **(H)**. ***p < 0.001, versus the DMSO group under normoxia. ns: not significant, ^#^ *p* < 0.05, ^##^ *p* < 0.01, ^###^ *p* < 0.001. versus the respective DMSO group under normoxia or hypoxia **(J)** Flow cytometry plots show the expression of CD235a and CD71 in HSPCs-differentiated erythrocytes treated the same as **(H)** and **(I)**. The graph on the right shows the percentage of CD71^+^/CD235a^+^ cells. **p < 0.01, versus the DMSO group under normoxia. ^###^ *p* < 0.001. versus the respective DMSO group under normoxia or hypoxia **(K)** May-Grunwald Giemsa staining of HSPCs-differentiated erythrocytes which were treated with DMSO or Baf A1 (20 nM) under normoxia or hypoxia for 11 days. Scale Bar = 20 μm. The right panel shows the morphologic classification. Cell types were determined by examining 200 cells per group and expressed as a percentage.

Considering that hypoxia can accelerate erythroid differentiation and enhance autophagy, we would like to know whether autophagy plays a role in erythroid differentiation promoted by hypoxia. We first treated K562-differentiated erythroid cells with Baf A1 for 3 days, and observed that the color of pellets was lightened by Baf A1 in both normoxia and hypoxia (Figure 3A), which means that blocking autophagy hampers the differentiation into erythroid cells. Simultaneously, the mRNA levels of *CD235a* and *HBG* were evidently decreased by Baf A1 at each indicated time point of differentiation, whether under normoxia or hypoxia (Figure 3B). The γ-globin protein levels were obviously reduced on the first, second and third day of differentiation into erythroid cells in both normoxia and hypoxia conditions, after autophagy was inhibited with Baf A1 as indicated by the accumulation of p62 and LC3-II (Figure 3C). In addition, flow cytometry analysis showed that the percentage of CD71^+^/CD235a^+^ was sharply reduced by Baf A1 treatment for 3 days under these two oxygen conditions (Figure 3D). The benzidine staining provided another evidence that inhibition of autophagy with Baf A1 resulted in a remarkable decrease in the percentage of benzidine-positive cells (Figure 3E). The above data consistently illustrate that blocking the autophagy process leads to serious obstacles in erythroid differentiation, and attenuates the accelerating effect of hypoxia on erythroid differentiation. Further, the expression of autophagy-related gene 5 (ATG5) or autophagy-related gene 7 (ATG7), which is involved in the initiation of autophagy, was silenced with siRNA to detect the perturbation of erythroid differentiation. Similarly, we found that knockdown of ATG5 or ATG7 significantly obstructed the differentiation into erythroid cells. In this case, the role of hypoxia in promoting erythroid differentiation was largely abated, which was manifested by a decrease in the protein and mRNA levels of CD235a and γ-globin under normoxia, as well as a decrease to their respective normoxia levels under hypoxia (Figures 3F,G).

In primary HSPCs, we first evaluated the effect of blocking autophagy with Baf A1 on the expression of β-globin and γ-globin in protein levels on the seventh day of erythroid differentiation. As expected, we discovered that the protein expression of β-globin and γ-globin was quite obviously decreased, especially under hypoxia, when autophagy was blocked as shown by the accumulation of p62 and LC3-II (Figure 3H). At the same time, we also examined the mRNA expression of *CD235a*, *HBG* and *HBB* after autophagy was blocked with Baf A1, and noted that their mRNA expression levels were substantially reduced without exception, particularly in hypoxia conditions (Figure 3I), potently supporting the results at the protein level. Flow cytometry analysis of the expression of CD235a and CD71 under the same treatment conditions as above showed that the autophagy inhibitor Baf A1 decreased the population of CD71^+^/CD235a^+^ in differentiated erythroid cells, and led to a greater reduction under hypoxia (Figure 3J). By May-Grunwald Giemsa staining to assess the process of erythroid differentiation, we provided another piece of evidence that Baf A1 deferred erythroid differentiation while inhibiting autophagy, and attenuated the acceleration of erythroid differentiation caused by hypoxia (Figure 3K). Collectively, our data show that autophagy plays a pivotal role in erythroid differentiation promoted by hypoxia.

### Suppression of mTORC1 under hypoxia is required for promoting autophagy and erythroid differentiation

**FIGURE 4:**
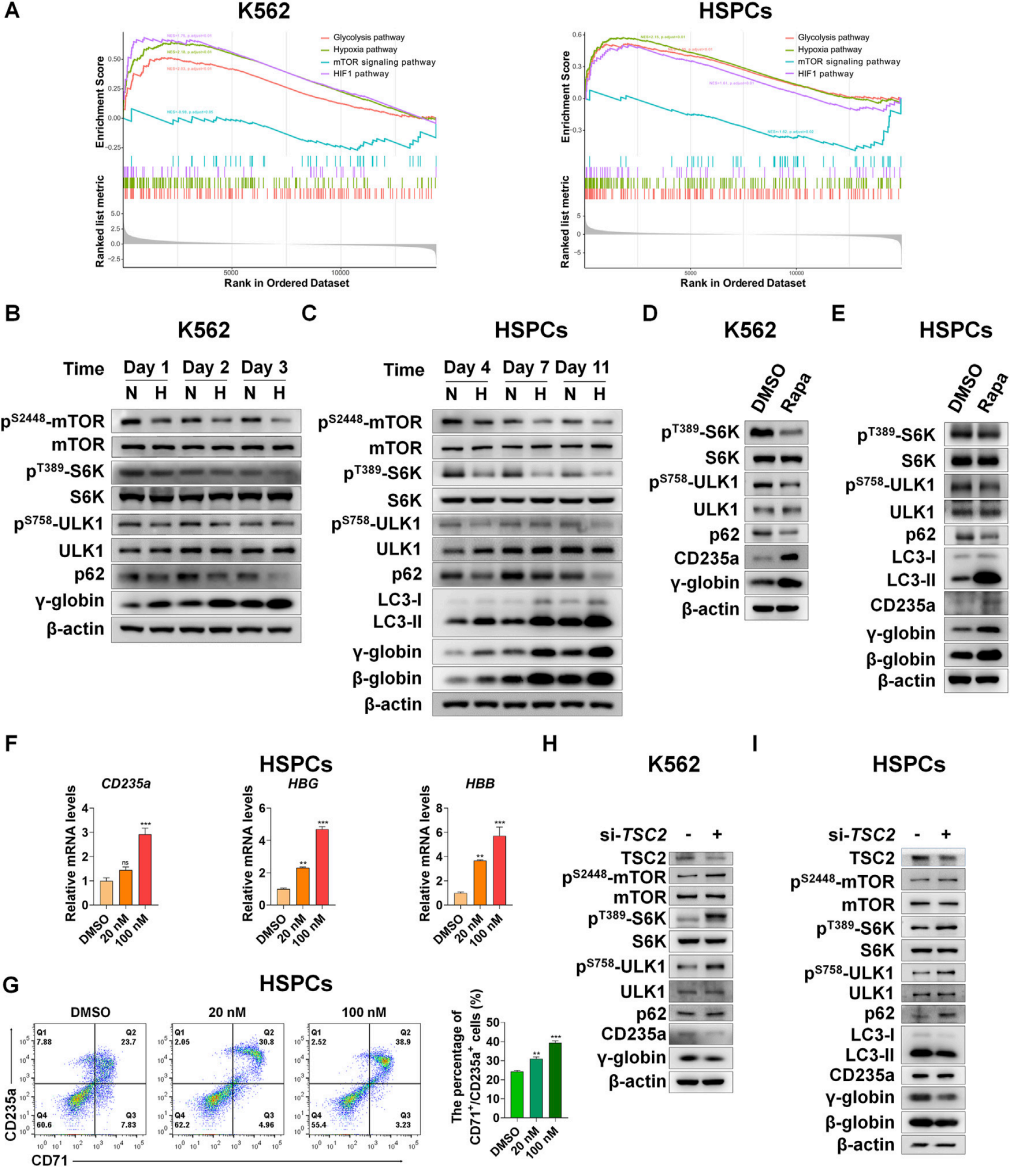
Suppression of mTORC1 is required for inducing autophagy and accelerating erythroid differentiation under hypoxia **(A)** After K562 cells (left) and HSPCs (right) differentiated erythrocytes were exposed to normoxia or hypoxia for 3 day and 7 days, respectively, RNA-seq was performed, and then GSEA was used to analyze the major pathways related to hypoxia. NES, normalized enrichment score **(B,C)** Western blot shows the effects of hypoxia on p62, LC3, γ-globin or β-globin protein levels and mTORC1 activity at different time points in the process of erythroid differentiation of K562 cells or HSPCs **(D,E)** Western blot analysis of the effects of Rapa (100 nM) on p62, LC3, CD235a, γ-globin or β-globin protein levels and mTORC1 activity in K562- or HSPCs-differentiated erythrocytes under normoxia **(F)** qRT-PCR analysis of the relative mRNA levels of CD235a, HBG and HBB after HSPCs were differentiated into erythrocytes which were treated with DMSO or different concentrations of Rapa for 3 days from the fourth day to the seventh day of differentiation under normoxia. ns, not significant, **p < 0.01, ***p < 0.001, versus the DMSO group **(G)** Flow cytometry plots show the expression of CD235a and CD71 in HSPCs-differentiated erythrocytes treated the same as **(F)**. The graph on the right shows the percentage of CD71^+^/CD235a^+^ cells. **p < 0.01, ***p < 0.001. versus the DMSO group **(H,I)** Western blot analysis of the effects of TSC2 knockdown on p62, LC3, CD235a, γ-globin or β-globin protein levels and mTORC1 activity in K562 cells- or HSPCs-differentiated erythrocytes under hypoxia.

To reveal the upstream regulators that promote autophagy in erythroid differentiation under hypoxia, we performed RNA-seq analysis after K562 or CD34^+^ cells were exposed to hypoxia during erythroid differentiation. We identified several signaling pathways by gene set enrichment analysis (GSEA) via comparing hypoxia with normoxia at day 3 or Day 7 of erythroid differentiation in K562 or HSPCs, respectively (Figure 4A). The results showed that the mRNAs upregulated in the hypoxia group were significantly enriched in the HIF-1, hypoxia and glycolysis pathways, simultaneously the mRNAs downregulated in the hypoxia group were enriched in the mTOR signaling pathway, and the pathways involved in HIF-1, hypoxia and glycolysis were negatively associated with the mTOR signaling pathway, both in K562 and HSPCs. The mTOR signaling that induces autophagy is directly controlled by the key switch, mammalian target of rapamycin complex 1 (mTORC1). It has been reported that mTORC1 is necessary for mitochondrial biosynthesis in erythroid differentiation (Liu et al., 2017). However, whether mTORC1 is involved in the induction of autophagy in erythroid differentiation, especially under hypoxia, is still unclear. In this work, we examined the dynamic changes in mTORC1 signaling, autophagy activity, and erythroid differentiation under normoxia and hypoxia via western blot analysis. In K562 cells, we found that the activities of mTOR, ribosomal S6 kinase 1 (S6K), and ULK1 decreased with differentiation time, which was indicated by the decrease in their respective phosphorylation levels, suggesting that mTORC1 signaling was gradually suppressed with the progress of erythroid differentiation. In the meantime, p62 and γ-globin protein levels decreased and increased with time, respectively, illustrating that the activity of autophagy increased with the differentiation into erythroid cells. Most notably, hypoxia caused these trends in advance (Figure 4B), which may be the reason why hypoxia accelerates erythroid differentiation. In HSPCs, we observed more clear changes in mTORC1 signaling and autophagy activity in the process of erythroid differentiation, and more effective impacts of hypoxia on them (Figure 4C).

To determine the role of mTORC1 signaling in regulating autophagy and thereby controlling erythroid differentiation, we used the mTORC1 inhibitor rapamycin (Rapa) to treat K562 cells and HSPCs during their differentiation into erythroid cells under normoxia conditions. As expected, we were excited to discover that when mTORC1 signaling was inhibited, autophagy activity and erythroid differentiation were enhanced (Figures 4D,E), which mimics the effect of hypoxia to some extent. Furthermore, the mRNA levels of *CD235a*, *HBG* or *HBB* and the percentage of CD71^+^/CD235a^+^ in HSPCs were all augmented in a dose-dependent manner by Rapa (Figures 4F,G), suggesting that the inhibition of mTORC1 activity is conducive to promoting the process of erythroid differentiation. On the contrary, increasing mTORC1 activity by silencing TSC2, which functions as a GTPase-activating protein (GAP) to inhibit the activation of mTORC1 mediated by accelerating GTP hydrolysis of Rheb (Yang et al., 2021), during the differentiation of these two types of cells into erythrocytes under hypoxia, resulted in a decrease in autophagy activity and erythroid differentiation (Figures 4H,I). Altogether, we provide strong evidence that mTORC1 is required for the control of autophagy, and the gradually decreased mTORC1 activity by hypoxia is conducive to a progressive increase in autophagy during erythroid differentiation.

### REDD1 acts as the upstream of mTORC1 to regulate autophagy and erythroid differentiation under hypoxia

**FIGURE 5:**
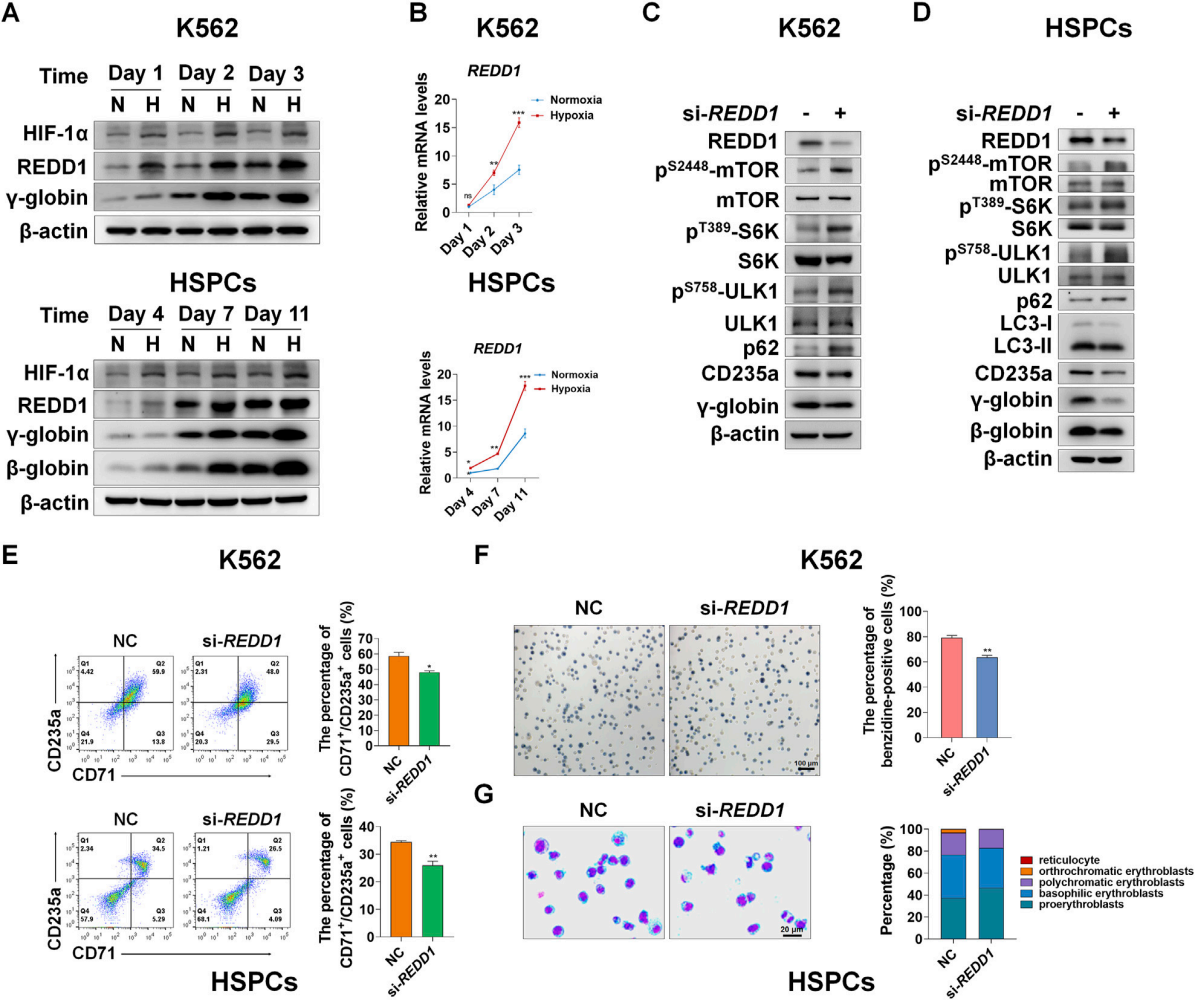
REDD1 upregulates autophagy through inhibition of mTORC1 signaling under hypoxia **(A)** Western blot analysis of HIF-1α and REDD1 protein expression at the indicated time points after K562 cells- and HSPCs-differentiated erythrocytes were exposed to normoxia or hypoxia. N: normoxia; H: hypoxia **(B)** qRT-PCR analysis of the relative mRNA levels of REDD1 in K562- and HSPCs-differentiated erythrocytes under normoxia or hypoxia. **p < 0.01, ***p < 0.001, versus the respective normoxia group at different time points **(C,D)** Western blot analysis of the effects of REDD1 knockdown on p62, LC3, CD235a, γ-globin or β-globin protein levels and mTORC1 activity in K562 cells- or HSPCs-differentiated erythrocytes cultured under hypoxia **(E)** Flow cytometry plots show the effects of REDD1 knockdown on CD71 and CD235a expression in K562 cells- and HSPCs-differentiated erythrocytes under hypoxia, respectively. The respective graph on the right shows the percentage of CD71^+^/CD235a^+^ cells. *p < 0.05, **p < 0.01, versus the NC group **(F)** Representative images of benzidine staining on the 3rd day of differentiation of K562 cells with or without REDD1 knockdown under hypoxia. Scale Bar = 100 μm. The graph on the right shows the percentage of benzidine-positive cells. **p < 0.01, versus the NC group **(G)** May-Grunwald Giemsa staining on the seventh day of differentiation of HSPCs shows the effect of REDD1 knockdown in hypoxia conditions on morphologic changes of erythrocytes. Scale Bar = 20 μm. The graph shows the percentage of different types of erythroblasts.

We next determined how mTORC1 is regulated by hypoxia during erythroid differentiation. Based on GSEA and heatmap analysis, we also found that genes related to the HIF-1 pathway were upregulated under hypoxia (Figure 4A, Supplementary Figure S6). Among them, REDD1 is well-known to play an essential role in suppression of mTORC1 activity in response to hypoxia (Brugarolas et al., 2004; Horak et al., 2010; Hoppe-Seyler et al., 2017; Sun and Yue, 2019; Gao et al., 2020; Shang et al., 2020). However, it has not yet been elucidated whether REDD1 induces autophagy by inhibiting mTORC1 signaling, thereby accelerating erythroid differentiation. First, to investigate the relationship between REDD1 and erythroid differentiation, we examined the changes in protein and mRNA levels of REDD1 as K562 or HSPCs were differentiated into erythrocytes under normoxia and hypoxia conditions, respectively. We noted that under normoxia, the expression levels of REDD1 protein and mRNA increased with erythroid differentiation, and compared with normoxia, hypoxia stimulated a further increase in REDD1 protein and mRNA levels, which was highly consistent with the changes in γ-globin or β-globin (Figures 5A,B), indicating that a higher level of REDD1 may be conducive to the acceleration of erythroid differentiation. Next, in order to gain insight into the role of REDD1 in regulating erythroid differentiation through the mTORC1-autophagy pathway, we silenced the expression of *REDD1* with siRNA under hypoxia, and then detected the effects of REDD1 knockdown on mTORC1 signaling, autophagy and erythroid differentiation. Western blot analysis showed that the silencing of REDD1 in K562 and HSPCs reduced the protein expression of CD235a, γ-globin or β-globin while activating mTORC1 activity and decreasing autophagy activity, manifested as an increase in the phosphorylation level of mTOR, S6K, ULK1 and an increase in p62 or a decrease in LC3-II (Figures 5C,D). In addition, we further confirmed the role of REDD1 in hypoxia-promoted erythroid differentiation by silencin*g* REDD1 in both K562 and HSPCs. We discovered that in the process of erythroid differentiation under hypoxia conditions, knockdown of REDD1 in K562 and HSPC significantly reduced the percentage of CD71^+^/CD235a^+^, as measured by flow cytometry analysis (Figure 5E). Meanwhile, the benzidine staining in hemin-induced K562 cells showed that silencing REDD1 led to an obvious decrease in the percentage of benzidine-positive cells (Figure 5F). Likewise, the knockdown of REDD1 during the differentiation of HSPCs into erythrocytes caused a serious delay in erythroid development, which was assessed by May-Grunwald Giemsa staining (Figure 5G). Overall, these data provide sufficient evidence that REDD1 plays a crucial role in erythroid differentiation, especially in the case of hypoxia accelerating erythroid differentiation, which may be achieved by suppressing the activity of mTORC1 to enhance autophagy.

## Discussion

**FIGURE 6:**
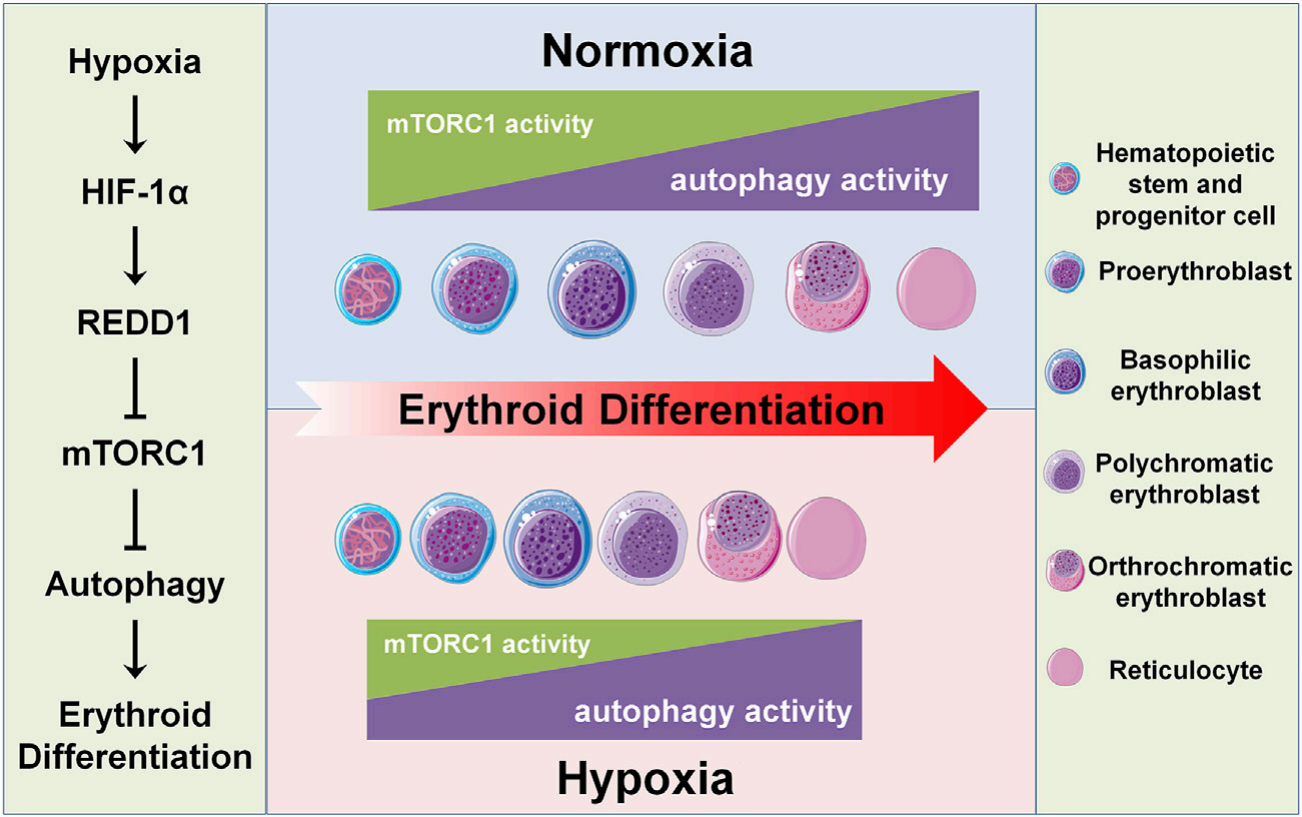
Schematic diagram of hypoxia accelerating erythroid differentiation in HSPCs. The left panel shows the underlying mechanism of hypoxia accelerating erythroid differentiation. Under hypoxia, HIF-1 as a master transcription factor transcriptionally activates its target gene REDD1, which in turn inhibits mTORC1 activity, thereby promoting autophagy activity and hence accelerating erythroid differentiation. The central panel shows the pattern of erythroid differentiation under the control of mTORC1 and autophagy in normoxia and hypoxia conditions. Under normoxia, with the process of erythroid differentiation, the activity of mTORC1 gradually decreases and the autophagy activity increases concomitantly; under hypoxia, these trends are enhanced, thus accelerating the erythroid differentiation. The right panel shows the icons.

It has not been elucidated how the differentiation of HSPCs into erythroid cells is regulated by a hypoxic niche. This study clarified that hypoxia can accelerate erythroid differentiation by promoting autophagy. The role of autophagy in erythroid differentiation, especially in a hypoxic niche, remains quite unclear except for the mitochondrial autophagy (termed as mitophagy) at the terminal stage of erythroid differentiation. Here, we reveal an underlying mechanism by which the erythroid differentiation accelerated by hypoxia can be achieved by the HIF-1/REDD1/mTORC1/autophagy pathway. Meanwhile, we also elucidate a novel role of mTORC1 in dynamic controlling erythroid differentiation by regulation of autophagy, in addition to by regulation of mitochondrial biosynthesis. We additionally find a new regulatory pattern of erythroid differentiation, that is, with the differentiation of HSPCs into mature erythrocytes, autophagy activity gradually increases with the gradual decrease of mTORC1 activity, and hypoxia accelerates this process (Figure 6).

The effect of hypoxia on the differentiation of HSPCs into erythroid cells is poorly understood, although increasing evidence supports that the quiescence and self-renewal of HSPCs can be maintained under a hypoxic niche (Yin and Li, 2006; Arai and Suda, 2008; Liu et al., 2019; Balise et al., 2020). There are few and contradictory studies on the effect of hypoxia on erythroid differentiation. A previous study showed that hypoxia promoted the transcription of HBG, but inhibited the transcription of HBB (Rogers et al., 2008). A recent study showed that hypoxia increased the proportion of CD235^+^ cells and promoted enucleation, but did not affect the expression of hemoglobin (Bapat et al., 2021). None of these studies yielded consistent results for elucidating the effect of hypoxia on erythroid differentiation, so that their evidence was insufficient to support their respective conclusions. Our data consistently demonstrates that hypoxia can promote erythroid differentiation. Importantly, we found that hypoxia accelerated the differentiation of HSPCs into erythroid cells, leading to erythroid maturation in advance.

It has been well documented that autophagy is necessary for the clearance of mitochondria at the terminal stage of erythroid differentiation (Schweers et al., 2007; Chen et al., 2008; Kundu et al., 2008; Sandoval et al., 2008; Mortensen et al., 2010; Davuluri et al., 2016; Grosso et al., 2017; Kuhikar et al., 2020; Moras et al., 2020; Moras et al., 2021). However, it is still unclear whether autophagy is also involved in other stages of erythroid differentiation, what role autophagy plays in them, and how hypoxia regulates autophagy during these stages of erythroid differentiation. The present study provides evidence to clarify these issues. We first demonstrated that hypoxia greatly accelerated erythroid differentiation using K562 cell line and primary HSPCs derived from umbilical cord blood. To investigate whether autophagy mediates the erythroid differentiation accelerated by hypoxia, we examined the effect of hypoxia on autophagy. In hemin-induced K562 cells, we did not observe the obvious effect of hypoxia on autophagy at the beginning, since LC3-II levels were not increased under hypoxia. However, p62 levels were reduced by hypoxia, indicating that hypoxia induced autophagy. It was not until later administration of the autophagy inhibitor Baf A1 resulted in the accumulation of more LC3-II under hypoxia than under normoxia that we were convinced that autophagy was actually enhanced by hypoxia. In HSPCs, we observed that in the process of HSPC differentiation into erythrocytes, hypoxia obviously promoted autophagy, as manifested by an increase in LC3-II and a decrease in p62 via western blot analysis, as well as the increased autophagosomes detected by TEM. To gain insight into the role of autophagy in erythroid differentiation under hypoxia, we next analyzed the effect of inhibiting autophagy on erythroid differentiation. Our data demonstrated that the accelerated erythroid differentiation by hypoxia was abrogated by inhibition of autophagy, indicating that autophagy is necessary and beneficial for accelerating erythroid differentiation under hypoxia. We speculate that the autophagy promoted by hypoxia may be responsible for the acceleration of erythroid differentiation, that is, the enhanced autophagy under hypoxia provides a rapid turnover of cellular components for cells, thereby accelerating erythroid differentiation.

Next, we uncovered a signaling pathway to regulate autophagy under hypoxia. We identified by GSEA the mTOR signaling pathway which is involved in the regulation of autophagy. mTORC1 is a control hub in the cell that integrates many processes, including promoting cell growth and suppressing autophagy through phosphorylation of a variety of substrates, such as S6K, autophagy activating protein ULK1, etc (Kim et al., 2011; Noda, 2017; Saxton and Sabatini, 2017; Park et al., 2018). In the present study, we found that hypoxia decreased the activity of mTORC1 gradually during erythroid differentiation, while correspondingly enhanced autophagy and accelerated erythroid differentiation. In normoxia, the specific inhibition of mTORC1 with rapamycin also enhanced autophagy and accelerated erythroid differentiation, mimicking the effect of hypoxia. In contrast, the specific activation of mTORC1 by silencing TSC2 in hypoxia suppressed autophagy and delayed erythroid differentiation. Unexpectedly, we found that the application of Rapa at the initial stage of differentiation inhibited rather than promoted erythroid differentiation (data not shown). It was not until later we delayed the addition of Rapa that the role of hypoxia in erythroid differentiation was successfully simulated. This finding is consistent with a recent study (Liu et al., 2020). In that study, they demonstrated that Rapa significantly suppressed erythroid colony formation in the commitment phase of erythropoiesis, whereas during the maturation phase of erythropoiesis, mTOR inhibition dramatically promoted enucleation and mitochondrial clearance by enhancing autophagy. In our study, we also noticed that in the process of erythroid differentiation, with the gradual decrease of mTORC1 activity, the autophagy activity gradually increased. Previous studies have shown that mTORC1 and autophagy are required for mitochondrial biosynthesis and elimination (Schweers et al., 2007; Sandoval et al., 2008; Liu et al., 2017; Summer et al., 2019; Xu et al., 2020), respectively. Our data and these reports allow us to infer that a higher mTORC1 activity is necessary for mitochondrial biosynthesis at the early stage of erythroid differentiation, and a higher autophagy activity is necessary for mitochondrial elimination at the terminal stage of erythroid differentiation. During this period, the gradual decrease in mTORC1 activity and the gradual increase in autophagy activity are conducive to reducing the synthesis of unwanted proteins and eliminating unwanted organelles, proteins, lipids, etc. Hypoxia can accelerate erythroid differentiation precisely because it augments the inhibition of mTORC1, which in turn enhances autophagy.

How does hypoxia inhibit the activity of mTORC1? The regulation of mTORC1 by hypoxia involves two pathways mediated by REDD1 and BNIP3, both of which are downstream target genes of HIF-1. In REDD1-mediated pathway, under normoxia, the phosphorylated TSC2 by the AKT kinase is translocated from lysosomal membrane to cytoplasm via binding of 14-3-3 protein, and therefore the inhibition of Rheb is relieved and mTORC1 is activated (Manning et al., 2002; Cai et al., 2006); under hypoxia, 14-3-3 binds to REDD1 upregulated by HIF-1 to reduce its binding to TSC2, thus releasing TSC2 to suppress mTORC1 (Brugarolas et al., 2004; DeYoung et al., 2008; Horak et al., 2010). In BNIP3-mediated pathway, under normoxia, the Rheb/mTORC1 pathway is activated; under hypoxia, Rheb interacts with BNIP3 upregulated by HIF-1 to reduce its association with mTORC1, thereby decreasing the activity of mTORC1 (Li et al., 2007; Lin et al., 2014). Considering that the silencing of TSC2 leads to a concomitant decrease in autophagy and erythroid differentiation, we investigated whether REDD1, as the upstream of TSC2, is involved in the regulation of autophagy during erythroid differentiation under hypoxia. Our results showed that the expression level of REDD1 was negatively correlated with the activity of mTORC1, but positively correlated with autophagy and erythroid differentiation. Most excitingly, silencing REDD1 under hypoxia delayed erythroid differentiation while activating mTORC1 and suppressing autophagy. These data suggest that REDD1 is required for increased autophagy and erythroid differentiation under hypoxia. Whether other pathways are also involved in the regulation of mTORC1 and thus autophagy to govern erythroid differentiation under hypoxia, such as BNIP3- or AMPK- mediated signaling pathways, cannot be ruled out in this study.

In summary, this work illustrates the importance of hypoxia in accelerating erythroid differentiation, and points out that autophagy is necessary for this effect of hypoxia, which has not been described previously. In the aspect of mechanism, the HIF-1/REDD1/TSC2/mTORC1 signaling axis has been identified as a regulatory pathway by which autophagy is enhanced under hypoxia, thereby accelerating erythroid differentiation. Moreover, we discovered a new regulatory pattern that in the process of erythroid differentiation, mTORC1 activity gradually decreases and autophagy activity increases progressively. Therefore, simultaneous analysis of mTORC1 and autophagy is essential for the determination of erythroid differentiation. Taken together, our findings can not only provide regulatory means for erythroid differentiation, but also contribute intervention strategies for the diagnosis and therapy of correlated erythroid diseases.

## Experimental procedures

### Cell culture

Human umbilical cord blood (UCB) was obtained from donors undergoing normal full-term deliveries after informed consent in accordance with procedures approved by the Institutional Research Ethics Committee of our institute. Primary human CD34^+^ cells were obtained from magnetically-sorted mononuclear samples of UCB as previously described (Zheng et al., 2014) and were frozen down after isolation. Briefly, Mononuclear cells were prepared by HISTOPAQUE (Sigma Aldrich, 10771). CD34^+^ cells were enriched using human CD34 MicroBead Kit (Miltenyi Biotec, 130-046-702). The CD34^+^ cells were maintained, expanded and differentiated as previously described with minor modifications (Zheng et al., 2014). Briefly, cells were thawed and washed into IMDM (Sigma Aldrich, 16529) with 20% FBS and 2 mM l-Glutamine (Sigma Aldrich, G7513), and then seeded for expansion in StemSpan SFEM Medium (STEMCELL Technologies, 09650) supplied with 50 ng/ml rhSCF (PeproTech, 300-07), 100 ng/ml rhTPO (PeproTech, 300-18), 100 ng/ml rhFlt3-Ligand (PeproTech, 300-19), 50 ng/ml rhIL-3 (PeproTech, 200-03) and 100 ng/ml rhIL-6 (PeproTech, 200-06). Cells were incubated at 37°C with 5% CO_2_ and maintained in the expansion medium at a density of 0.3 × 10^6^ to 1 × 10^6^ cells per milliliter with media changes every 2–3 day. *Ex vivo* erythroid maturation of human CD34^+^ cells was performed using a two-phase liquid culture system. On day 7, cells were centrifuged and transferred into EDM (erythroid differentiation medium) comprising IMDM medium, 3 U/ml rhEPO (PeproTech, 100-64), 2 mM l-Glutamine, 2% PS, 330 μg/ml human holo-Transferrin (Sigma Aldrich, T4132), 10 μg/ml human insulin (Sigma Aldrich, I9278), 2 U/ml heparin (STEMCELL Technologies, 07980) and 5% inactivated human serum (Sigma Aldrich, H3667) supplemented with 100 ng/ml rhSCF, 5ng/ml rhIL-3 and 1 μM dexamethasone (Sigma Aldrich, D4902). On day 14, cells were centrifuged and transferred into EDM supplemented with 100 ng/ml rhSCF. Cells were maintained in differentiation medium for indicated times at a density of 0.3–1 × 10^6^ cells/ml by supplementing cultures every few days with fresh media. K562 cells were maintained in RPMI 1640 Medium containing 10% fetal bovine serum (FBS), and were induced to differentiate into erythroid cells with the supplement of Hemin (30 μmol/L).

### Hypoxia and reagent treatment

For hypoxia treatment, cells were incubated in an O_2_/N_2_/CO_2_ incubator (Thermo Fisher Scientific, Marietta, OH, United States; 3131) and exposed to 3% O_2_ for the indicated time periods. For reagent treatment, DMSO or particular chemicals were added to the differentiation medium, which were used as the control group and the experimental group, respectively. Baf A1 (HY-100558) and Rapa (HY-10219) were purchased from MedChemExpress (Monmouth Junction).

### Small interfering (si) RNA transfection

Transfection was performed 30 min after cells were plated at a density of 3×10^5^ cells/ml. Negative control (si-NC) and siRNA targeting the indicated mRNA (Supplementary Table S1) were purchased from RiboBio (Guangzhou, China). Cells were transiently transfected with si-NC or si-*ATG5*/si-*ATG7*/*REDD1*/*TSC2* at a final concentration of 100 nM using Lipofectamine 2000 (Thermo Fisher Scientific, 11668-019) according to the manufacturer’s instructions. For K562 cells, the relevant analysis was performed on the third day after transfection that was conducted on the first day of differentiation; For HSPCs, the relevant analysis was performed on the seventh day after transfection that was conducted on the fourth day of differentiation.

### Flow cytometry analysis

For staining, single-cell suspensions were incubated on ice with indicated antibodies in PBS. Cells were run on FACSAria II Cell Sorter (BD Biosciences) and analyzed using Flow Jo VX. FITC Mouse Anti-human CD71 (BD Biosciences, 555536) and anti-human CD235a APC (Thermo Fisher Scientific, 17-9987-41) were used for cell surface staining.

### Benzidine stain assay

Benzidine staining assay was performed as previously described (Fibach and Prus, 2005). Briefly, benzidine stock solution containing 0.5 M glacial acetic acid and 0.4% benzidine dihydrochloride (Sigma Aldrich, B3383) was mixed with PBS and 33% H_2_O_2_ at a 25:25:1 (v/v/v) ratio to prepare working solution. Cells were resuspended in PBS and incubated with benzidine working solution at a 100:1 (v/v) ratio at room temperature. After 3–5 min, the cells were examined using a light microscope (Nikon, ECLIPSE, Ti2-U). The benzidine-positive cells were blue.

### Giemsa stain assay

Giemsa stain assay was performed as previously described (Ma et al., 2020). Briefly, the cells were collected by centrifugation and resuspended in PBS at different times of differentiation. Then cells were cytocentrifuged onto glass slides and stained with Wright Giemsa Stain Kit (Solarbio, Beijing, China; G1021) at room temperature. Stained cells from each group were examined under a light microscope (Nikon, ECLIPSE, Ci-L).

### Immunofluorescence assay

For Immunofluorescence analysis, cells were centrifuged onto glass slides and fixed with 4% paraformaldehyde (PFA) in PBS for 10 min. Then, cells were incubated with 0.3% Triton X-100 in PBS for 10 min and blocked with 2% bovine serum albumin in PBS for 1 h, followed by overnight incubation at 4°C with primary antibodies (Supplementary Table S2). On the second day, cells were washed 3 times in PBS, 5 min each time, and corresponding secondary antibodies (Supplementary Table S2) were added and incubated for 1 h at room temperature, followed by incubation with Hoechst 33342 (1:1000; Solarbio, C0031) for 5 min at room temperature and rinsing 3 times before mounting on glass slides with VECTASHIELD antifade mounting medium (Wolcavi, H-1200). Images were captured by an inverted confocal microscope (UltraVIEW VOX, PerkinElmer, Waltham, Massachusetts, United States) using CY3/DAPI filters at 60X objective, and fluorescence quantification was performed using ImageJ.

### Transmission electron microscopy

For electron microscopy evaluation, cells were collected and washed with PBS after cells were exposed to normoxia or hypoxia for indicated times, and then fixed with 3% glutaraldehyde in 0.1 M PBS at 4°C for 2 h. The subsequent steps were performed as previously described (Verheije et al., 2008). TEM analysis was performed by using a transmission electron microscope (H-7650; HITACHI, Tokyo, Japan) at 80 kV.

### Real time quantitative polymerase chain reaction (RT-qPCR)

Total RNA was extracted from cells using RNApure Tissue/Cell Kit (DNase Ⅰ) (CWBIO, CW0560S). Next, 500 ng of total RNA was reverse transcribed into cDNA using the MonScript RTⅢ All-in-One Mix (BioPro, MR05001) according to the manufacturer’s instructions. RT-qPCR was performed with KAPA SYBR^®^ FAST (Roche, KK4601) under the following conditions: 5 min 20 s at 95°C, followed by 40 cycles (5 s at 95°C and 30 s at 60°C). Primers for qPCR were synthesized by Tianyi Huiyuan (Beijing, China) and listed in Supplementary Table S3. Each reaction was repeated in at least three independent experiments. The 2^−ΔΔCt^ method to calculate the relative expression levels (fold change) was used. Amplification of the housekeeping gene *ACTB* was used to normalize the amount of cDNA.

### Western blot analysis

Total protein was extracted from cells with RIPA Lysis Buffer (CWBIO, CW2333) containing a protease inhibitor cocktail (Roche, 04,693,132,001). Equal amounts of protein were separated on 8%–12% SDS-PAGE gels and transferred to PVDF membranes (Millipore, ISEQ00010), which were blocked with 5% skimmed milk for 1 h and incubated with primary antibodies (Supplementary Table S2) overnight at 4°C. This was followed by incubation with appropriate secondary antibodies (Supplementary Table S2) for 2 h at room temperature. Next, the protein bands were visualized using SuperSignal West Pico PLUS (Thermo Fisher Scientific, 34580). β-actin was used as the loading control. The quantitation of western blot is shown in supplementary figures (Supplementary Figure S1-S5).

### Quantification and statistical analysis

Data were represented as mean ± standard error of the mean of at least three independent experiments. Statistical significance differences were carried out using the unpaired two-sided Student’s t test, one-way analysis of variance (ANOVA) followed by Dunnet’s post hoc test and two-way analysis of variance (ANOVA) followed by sidak’s post hoc test, using the Microsoft Excel or GraphPad Prism 8.0.2. The levels of significance were indicated by ns, not significant; *p < 0.05, **p < 0.01, and ***p < 0.001. *p* < 0.05 was considered statistically significant.

## Data Availability

The original contributions presented in the study are publicly available. This data can be found here: https://www.ncbi.nlm.nih.gov/geo/query/acc.cgi?acc=GSE199778.
