# Supplementary material for: Autophagy regulated by the HIF/REDD1/mTORC1 signaling is progressively increased during erythroid differentiation under hypoxia
Source: Front Cell Dev Biol. 2022 Aug 24;10:896893. doi: 10.3389/fcell.2022.896893 (PMC9448881; doi:10.3389/fcell.2022.896893)
Supplement: Supplementary file 1 [file DataSheet1.docx]

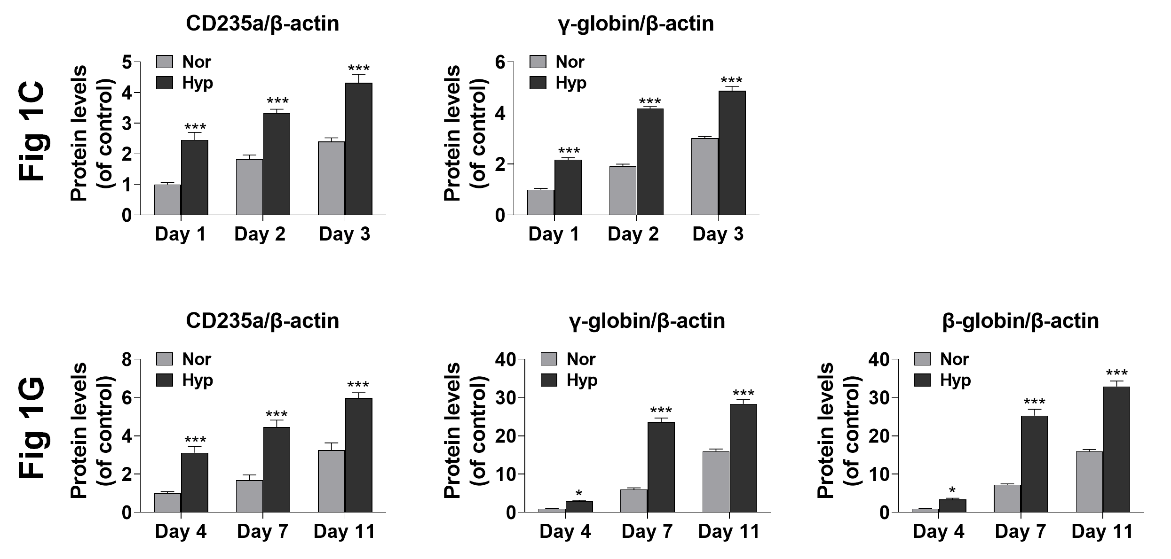


**Figure S1**. The quantitative analysis of western blot for Figure 1C and 1G. The relative protein expression of CD235a and γ-globin or β-globin at the indicated time points after K562 cells (**Fig 1C**) or HSPCs (**Fig 1G**) were exposed to normoxia or hypoxia. Nor: normoxia; Hyp: hypoxia. **P*<0.05, ****P*<0.001, versus the respective normoxia group.


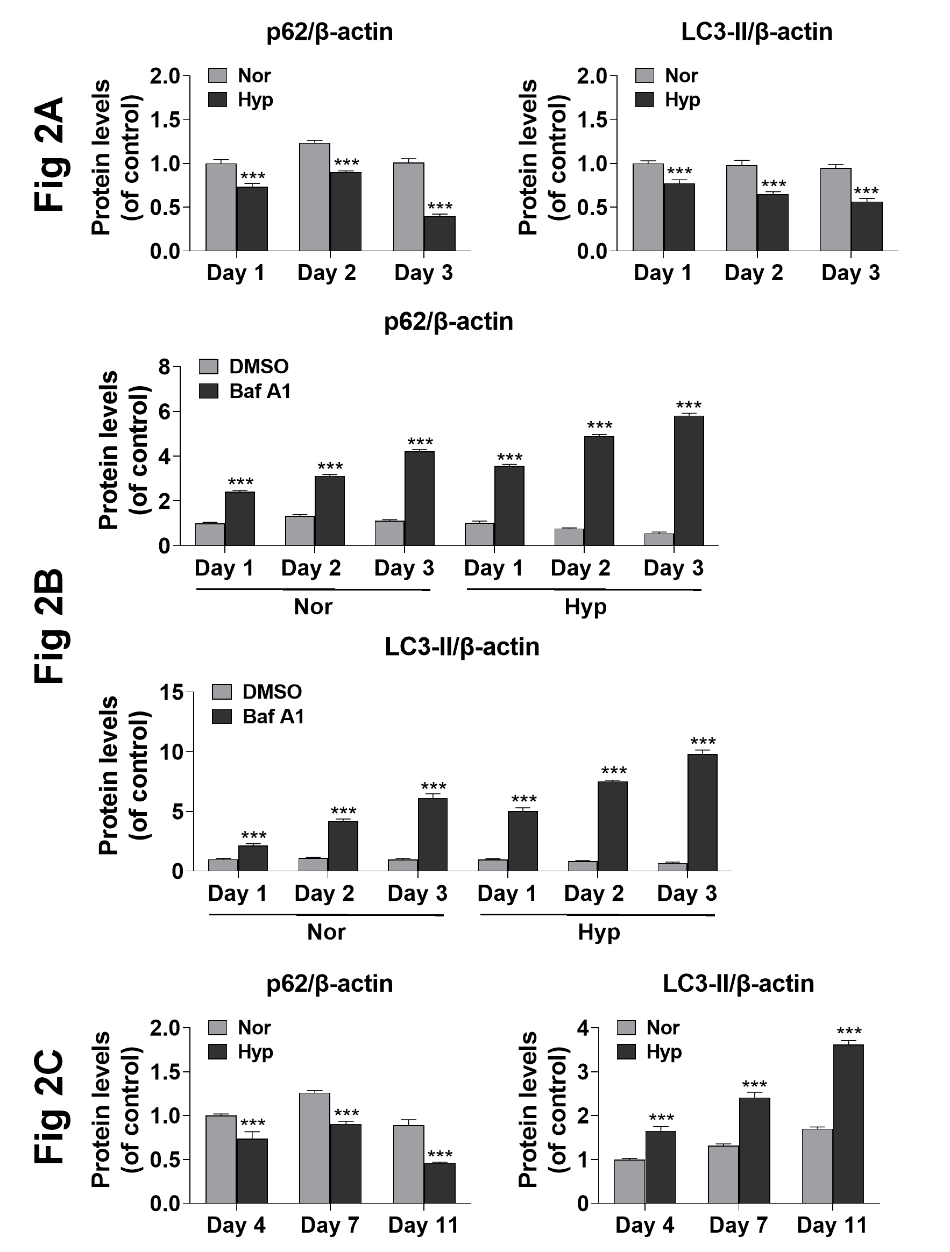


**Figure S2**. The quantitative analysis of western blot for Figure 2A, B, C. The relative protein expression of p62 and LC3-II at the indicated time points after K562 cells (**Fig 2A**) or HSPCs (**Fig 2C**) were exposed to normoxia or hypoxia. Nor: normoxia; Hyp: hypoxia. **P*<0.05, ****P*<0.001, versus the respective normoxia group. (**Fig 2B**) The relative protein expression of p62 and LC3 at the indicated time points after K562 cells treated with DMSO (-) as control or Baf A1 (+, 20 nM) were exposed to normoxia or hypoxia. Nor: normoxia; Hyp: hypoxia. ****P*<0.001, versus the DMSO control.


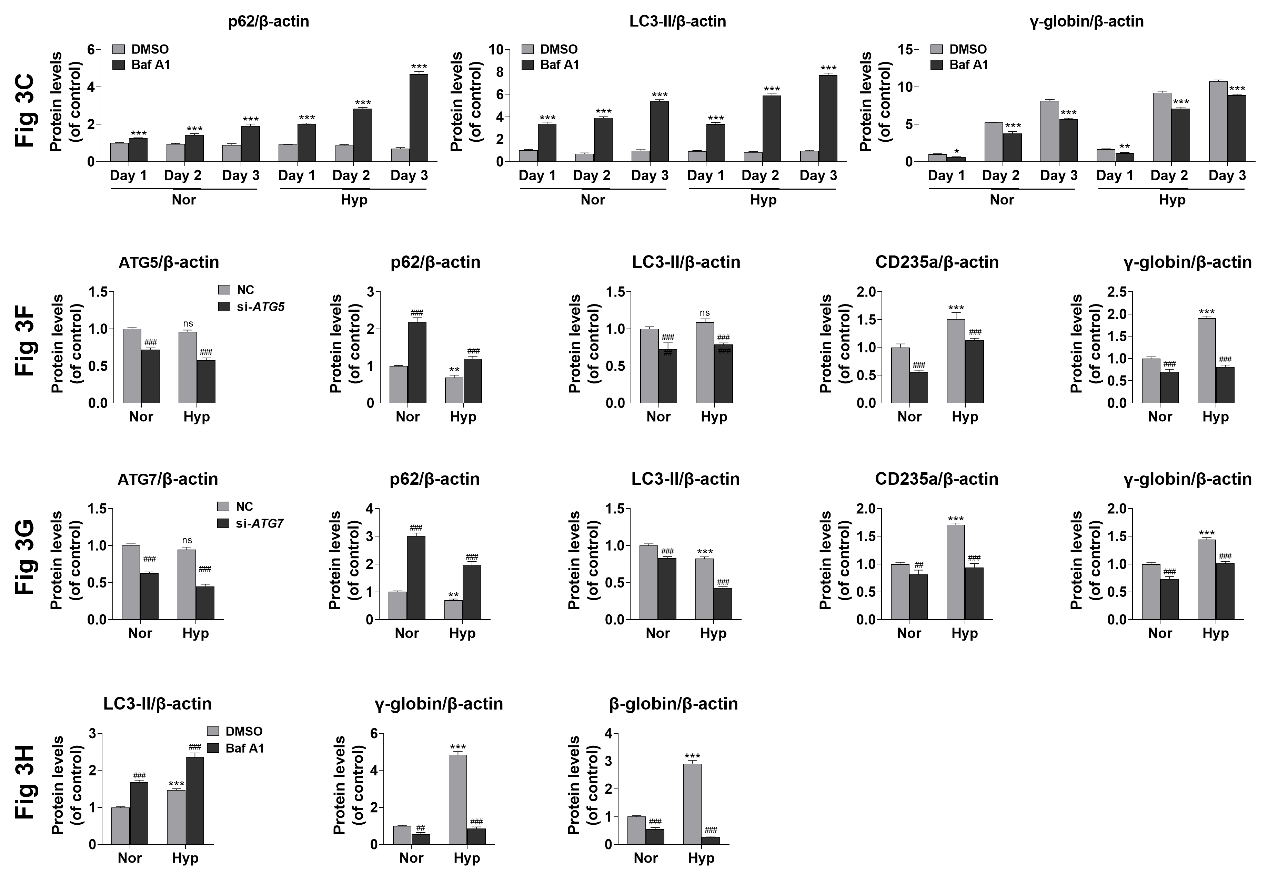


**Figure S3**. The quantitative analysis of western blot for Figure 3C, F, G, H. (**Fig 3C**) The relative protein expression of p62, LC3 and γ-globin at the indicated time points after K562 cells differentiated into erythrocytes were treated with DMSO (-) as control or Baf A1 (+, 20 nM) under normoxia or hypoxia. Nor: normoxia; Hyp: hypoxia. **P*<0.05, ***P*<0.01, ****P*<0.001, versus the DMSO control. (**Fig 3F, G**) The relative protein expression of ATG5, ATG7, p62, LC3, CD235a and γ-globin in differentiated K562 cells after they were transfected with scramble siRNA as negative control (NC) or si-*ATG5*/*ATG7* under normoxia or hypoxia for 3 days. Nor: normoxia; Hyp: hypoxia. ***P*<0.01, ****P*<0.001, versus the NC control; ^##^*P*<0.01, ^###^*P*<0.001, versus the negative control. (**Fig 3H**) The relative protein expression of LC3, γ-globin and β-globin in HSPCs-differentiated erythrocytes which were treated with DMSO or Baf A1 under normoxia or hypoxia for 7 days. Nor: normoxia; Hyp: hypoxia. ****P*<0.001, versus the respective normoxia group; ^##^*P*<0.01, ^###^*P*<0.001, versus the DMSO control.


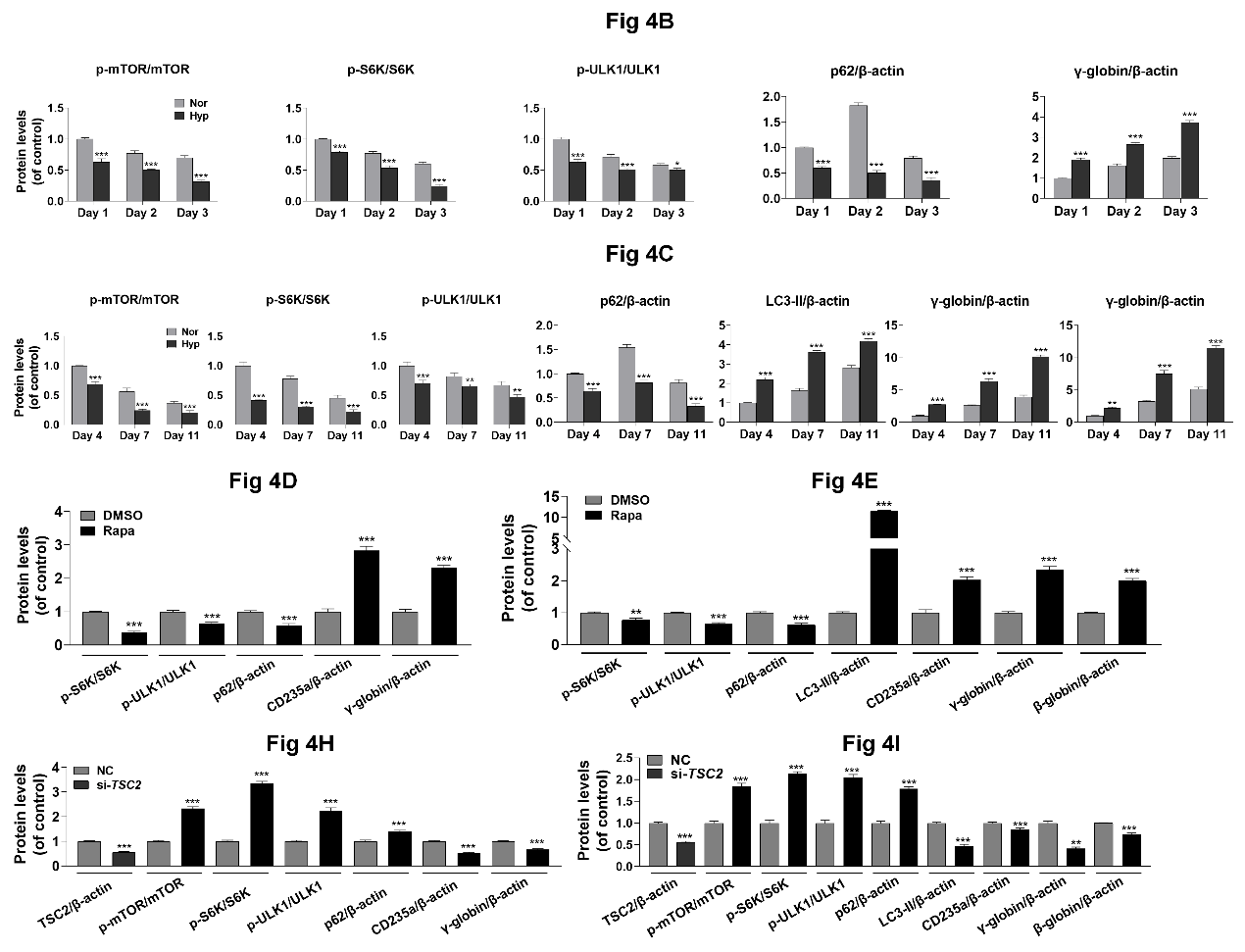


**Figure S4**. The quantitative analysis of western blot for Figure 4B, C, D, E, H, I. The relative protein expression of p62, LC3, γ-globin or β-globin protein levels and mTORC1 activity at different time points in the process of erythroid differentiation of K562 cells (**Fig 4B**) or HSPCs (**Fig 4C**). Nor: normoxia; Hyp: hypoxia. **P*<0.05, ***P*<0.01, ****P*<0.001, versus the respective normoxia group. The relative protein expression of p62, LC3, CD235a, γ-globin or β-globin and mTORC1 activity in K562 (**Fig 4D**)- or HSPCs (**Fig 4E**)-differentiated erythrocytes with or without rapamycin (Rapa) treatment under normoxia. ***P*<0.01, ****P*<0.001, versus the DMSO control. The relative protein expression of p62, LC3, CD235a, γ-globin or β-globin and mTORC1 activity in K562 cells (**Fig 4H**)- or HSPCs (**Fig 4I**)-differentiated erythrocytes with or without knockdown of TSC2 under hypoxia. ***P*<0.01, ****P*<0.001, versus the NC control.


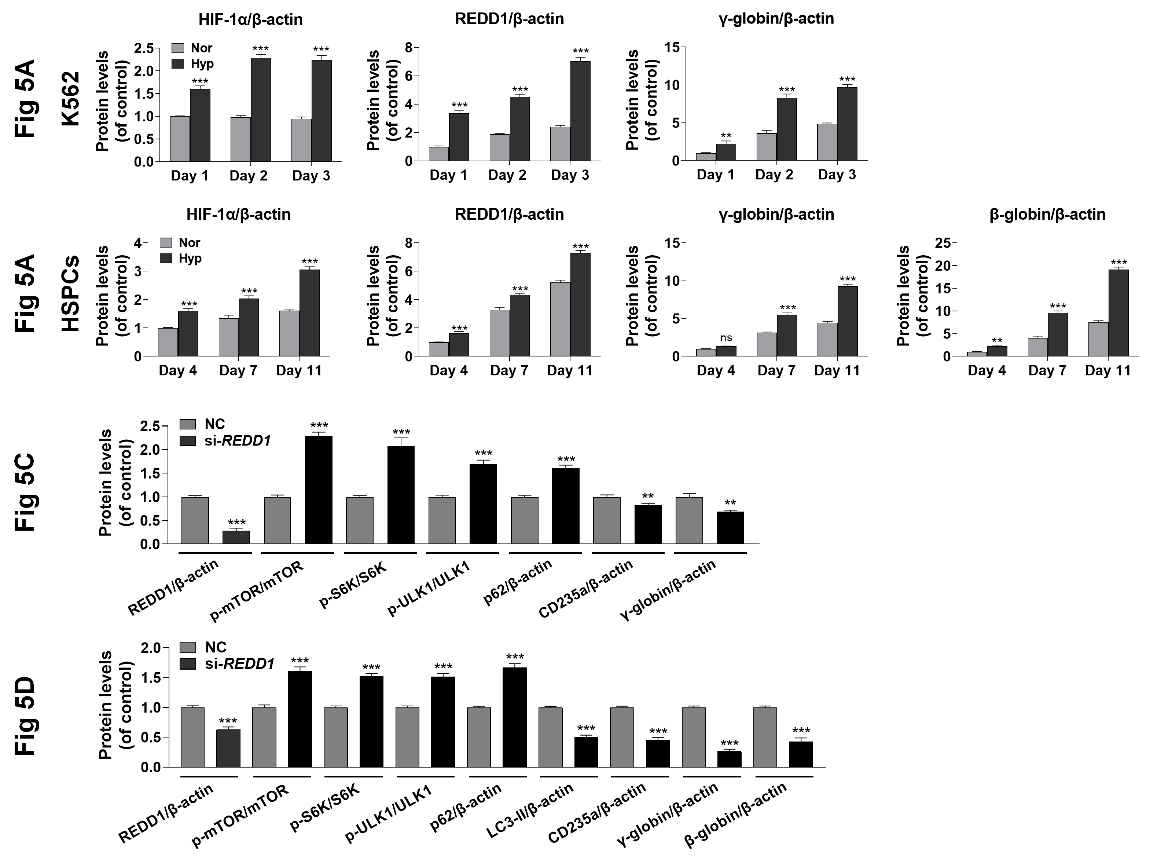


**Figure S5**. The quantitative analysis of western blot for Figure 5A, C, D. (**Fig 5A**) The relative protein expression of HIF-1α and REDD1 at the indicated time points after K562 cells (upper penal)- and HSPCs (lower penal)-differentiated erythrocytes were exposed to normoxia or hypoxia. N: normoxia; H: hypoxia. ***P*<0.01, ****P*<0.001, versus the respective normoxia group. The relative protein expression of p62, LC3, CD235a, γ-globin or β-globin protein levels and mTORC1 activity in K562 cells (**Fig 5C**)- or HSPCs (**Fig 5D**)-differentiated erythrocytes with or without knockdown of REDD1 under hypoxia. ***P*<0.01, ****P*<0.001, versus the NC control.


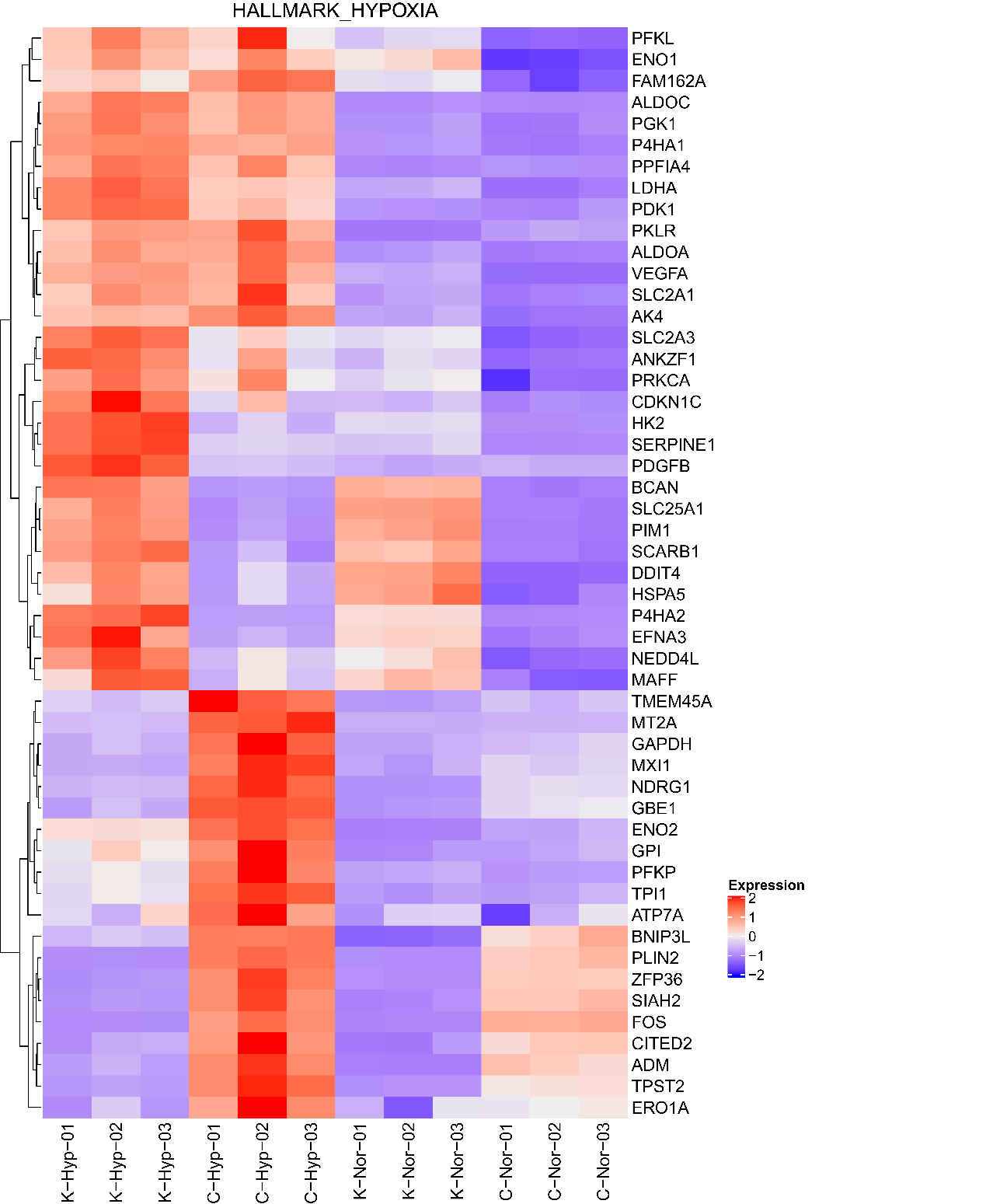


**Figure S6**. Expression heatmap for genes associated with hypoxia pathway. K: K562 cells, C: CD34^+^ cells. Nor: normoxia; Hyp: hypoxia.

**Supplementary Table S1.** The target sequence of siRNA

| Gene | Target Sequence |
| --- | --- |
| *ATG5*  *ATG7* | 5'-TGACGTTGGTAACTGACAA-3'  5'-GAACGAGTATCGGCTGGAT-3' |
| *REDD1* | 5'-CGGAACAGCTGCTCATTGA-3' |
| *TSC2* | 5'-GAACCTGATCTCCTATAGA-3' |

**Supplementary Table S2.** Antibodies for immunofluorescence assay and western blot analysis

| **Antibody** | **Application** | **Species** | **Source** | **Cat#** | **Dilution** |
| --- | --- | --- | --- | --- | --- |
| LC3B | IF/WB | Rabbit | Sigma Aldrich | L7543 | 1:200/1:2000 |
| anti-rabbit IgG | IF | Goat | CWBIO | CW0219 | 1:200 |
| γ-globin | WB | Mouse | Santa Cruz Biotechnology | sc-21756 | 1:2000 |
| β-globin | WB | Mouse | Santa Cruz Biotechnology | sc-21757 | 1:2000 |
| CD235a | WB | Rabbit | ABclonal | A1827 | 1:1000 |
| β-actin | WB | Mouse | ABclonal | AC004 | 1:2000 |
| HIF-1α | WB | Rabbit | Proteintech | 20960-1-AP | 1:1000 |
| ATG5 | WB | Rabbit | ABclonal | A19677 | 1:1000 |
| ATG7 | WB | Rabbit | Cell Signaling Technology | 8558S | 1:1000 |
| p62 | WB | Rabbit | Cell Signaling Technology | 5114S | 1:1000 |
| Phospho-mTOR (Ser2448) | WB | Rabbit | Cell Signaling Technology | 5536S | 1:1000 |
| mTOR | WB | Rabbit | Cell Signaling Technology | 2983T | 1:1000 |
| Phospho-p70 S6 Kinase (Thr389) | WB | Rabbit | Cell Signaling Technology | 9234S | 1:1000 |
| p70 S6 Kinase | WB | Rabbit | Cell Signaling Technology | 2708S | 1:1000 |
| Phospho-ULK1 (Ser757) | WB | Rabbit | Cell Signaling Technology | 14202S | 1:1000 |
| ULK1 | WB | Rabbit | Cell Signaling Technology | 8054S | 1:1000 |
| TSC2 | WB | Rabbit | Cell Signaling Technology | 4308S | 1:1000 |
| REDD1 | WB | Rabbit | Proteintech | 10638-1-AP | 1:1000 |
| anti-rabbit IgG | WB | Goat | Cell Signaling Technology | 7074P2 | 1:2000 |
| anti-mouse IgG | WB | Horse | Cell Signaling Technology | 7076P2 | 1:2000 |

**Supplementary Table S3.** The squences of primers for real-time quantiative polymerase chain reaction analysis

| Gene | Sequence |
| --- | --- |
| *HBG* | Forward: 5’-ACTCGCTTCTGGAACGTCTG-3’ |
|  | Reverse: 5’-CCTCTGGGTCCATGGGTAGA-3’ |
| *HBB* | Forward: 5’-AATTCACCCCACCAGTGCAG-3’ |
|  | Reverse: 5’-GGCAGAATCCAGATGCTCAAG-3’ |
| *CD235a* | Forward: 5’-ACAACTTGCCCATCATTTCTCTG-3’ |
|  | Reverse: 5’-TCAGTCGGCGAATACCGTAAG-3’ |
| *REDD1* | Forward: 5’-TGGGCAAAGAACTACTGCG-3’ |
|  | Reverse: 5’-AGAGTTGGCGGAGCTAAACAG-3’ |
| *ACTB* | Forward: 5’-GGATTCCTATGTGGGCGACGA-3’ |
|  | Reverse: 5’-GCGTACAGGGATAGCACAGC-3’ |
